# Specificity of Amino Acid Profiles Produced in Soybean Fermentations by Three *Bacillus* spp.

**DOI:** 10.4014/jmb.2411.11038

**Published:** 2024-12-02

**Authors:** Sumin Seo, Do-Won Jeong, Sooyoung Sul, Jong-Hoon Lee

**Affiliations:** 1 Department of Food Science and Biotechnology, Kyonggi University, Suwon 16227, Republic of Korea; 2 Department of Food and Nutrition, Dongduk Women’s University, Seoul 02748, Republic of Korea; 3 Division of Sports Science, Kyonggi University, Suwon 16227, Republic of Korea

**Keywords:** *Bacillus subtilis*, *Bacillus velezensis*, *Bacillus licheniformis*, amino acid profile, proteolytic activity, salt tolerance

## Abstract

We compared the salt tolerance and proteolytic activity of 120 strains of each of *Bacillus subtilis*, *Bacillus velezensis*, and *Bacillus licheniformis*. Most *B. subtilis* strains exhibited growth in 12% (w/v) NaCl and showed proteolytic activity in 10% or 11% NaCl. The majority of *B. velezensis* strains grew in 14% NaCl and showed proteolytic activity in 12% or 13% NaCl. Most *B. licheniformis* strains grew in 14% NaCl and exhibited proteolytic activity in 5%–7% NaCl. We selected nine representative strains of each species based on their proteolytic activities and analyzed the free amino acid (FAA) profiles produced by culture of the bacteria on soybean. Statistical analyses of the 22 FAAs quantified in the cultures revealed clustering of FAA production profiles at the species level. The FAA production profiles of *B. subtilis* and *B. velezensis* were similar, and both differed from that of *B. licheniformis*. These trends persisted in cultures containing 7% NaCl. These results suggest that FAA production profiles are characteristic of each *Bacillus* species. Specifically, in soybean cultures compared with uninoculated soybeans, *B. subtilis* increased the amounts of leucine and phenylalanine; *B. velezensis* increased the amounts of leucine, phenylalanine, and tyrosine; and *B. licheniformis* increased the amounts of alanine, glutamic acid, tyrosine, and ornithine, and dramatically decreased the amount of arginine. The proteolytic activity of *B. velezensis* strains correlated with the quantity of FAAs in their soybean cultures. Considering its salt tolerance and proteolytic activity, *B. velezensis* showed high potential for contributing to the ripening of high-salt fermented soybean foods. Our results regarding the specific production of amino acids at the species level and correlations between proteolytic activities and produced amino acid quantities will facilitate the determination and selection of target strains for functional *Bacillus*-fermented foods.

## Introduction

The characteristics of *Bacillus* spp. including endospore formation, diversity in physiological properties, as well as capacity to produce numerous antimicrobial compounds make them ubiquitous in nature. They are easily found in a wide range of foods, both as naturally occurring contaminants and as intentional additives. Because of their prevalence and enzyme-producing ability, *Bacillus* spp. are common fermentative bacteria in traditional fermented foods, especially in legume-based fermented foods of Asia and Africa [1].

Soybeans (members of the legume family) are one of the most significant and versatile sources of plant-based protein. They have been consumed for centuries, especially in East Asian cultures, and are the foundation of many plant-based protein products [2]. Fermentation is a significant processing technology for soybean in terms of enhancement of nutritional value, protein and amino acid transformation, flavor and texture development, and preservation [3]. Several studies, including microbial community analyses, have been performed to establish a robust scientific foundation for accelerated ripening, quality assurance, and flavor enhancement in the commercial production of fermented soybean foods and to suggest possible directions for future starter culture design for soybean fermentation [4]. *Bacillus* spp., mostly members of the *B. subtilis* group including *B. amyloliquefaciens*, *B. licheniformis*, *B. pumilus*, *B. subtilis*, and *B. velezensis*, have been identified as a populous bacterial group in fermented soybean foods. On the basis of their high rate of detection, *B. subtilis*-group members have been considered as food fermentation starter candidates. Numerous *Bacillus* starter candidates have been applied in food manufacturing processes and have been reported to achieve their intended purposes [5-8]. However, strain-level studies on *Bacillus* starter implementation have not provided sufficient information to determine the relevance of candidates for the development of particular fermented foods.

Fermented soybean foods consumed in Korea can be classified into two types based on salt concentrations. One type is manufactured without the addition of salt, while the other is produced under high-salt conditions (>12%NaCl, w/w). The proteolytic activity of *Bacillus* starters is essential in soybean fermentation, facilitating the breakdown of soy proteins into smaller peptides and amino acids. *Bacillus* spp. can thrive and maintain their proteolytic function at low salt concentrations, but their growth and enzymatic activity may be compromised at high salt concentrations. Here, we compared the salt tolerance and proteolytic activities of 120 strains of each of *B. subtilis*, *B. velezensis*, and *B. licheniformis*, which are frequently identified in Korean fermented soybean foods, and selected representatives of each *Bacillus* species showing particular characteristics. Then, we analyzed the free amino acid (FAA) profiles produced by the selected strains in soybean cultures to predict species-specific properties for amino acid production in fermented soybean foods. To the best of our knowledge, this study is the first to compare the amino acid-producing characteristics of *Bacillus* at the species level.

## Materials and Methods

### Bacillus Strains and Cultures

The *Bacillus* strains in this study were isolated from Korean fermented soybean foods. They were isolated in our previous studies, or kindly provided by the Korea Food Research Institute (http://www.kfri.re.kr), the Korean Agricultural Culture Collection (KACC; http://genebank.rda.go.kr/), or the Microbial Institute for Fermentation Industry (MIFI; http://www.mifi.kr/). Their taxonomic identities were confirmed by analysis of sequences of the housekeeping gene *gmk* (encoding guanylate kinase), and *B. licheniformis* strains were reconfirmed by gene sequence analysis of *spo0A* (encoding stage 0 sporulation protein A) [9, 10]. The strains were mainly cultured on Difco tryptic soy-agar (TSA; BD Diagnostic Systems, USA) and in Difco tryptic soy broth (TSB; BD Diagnostic Systems) at 37°C for 24 h.

### Assessment of Salt Tolerance and Proteolytic Activity

Salt tolerance of strains was determined by growth on TSA containing NaCl at a final concentration of <15% (w/v) with incubation at 37°C for 4 days. Proteolytic activity was determined on TSA supplemented with 2% (w/v) skim milk. The maximum NaCl concentration that allowed proteolytic activity was determined by the addition of NaCl at a final concentration of <14% (w/v) to the proteolytic activity test medium with incubation at 37°C for 4 days. Test strains were incubated in TSB to an optical density of 0.5 at 600 nm and 1 μl of this suspension was inoculated onto plates. Experiments were performed at least three times on separate days. Semi-quantitative determination of proteolytic activity used the proteolytic index (PI) value, which was determined from the ratio between the sizes (in cm) of the clear zone and colony developed on proteolytic activity test agar plates after 6 h of incubation at 37°C followed by incubation at 4°C for 1 day. The PI value applied in this study was modified from a method used for fungal extracellular protease determination [11].

### Preparation of *Bacillus* Strain-Inoculated Soybean Culture Samples

Soybeans (*Glycine max* L.; Korean Bactae) were washed and soaked in distilled water for 18 h at room temperature, and then strained to remove residual water. After dehulling, water-absorbed soybeans were ground using a blender and mixed thoroughly with distilled water to achieve a total solid content of 5% (w/w). One hundred milliliters of the soybean suspension were bottled in 250-ml reagent bottles and then autoclaved for 20 min at 121°C. Respective logarithmic-phase *Bacillus* cells cultured in TSB were inoculated into the sterilized soybean suspension at 5 × 10^5^ colony-forming units/g then mixed thoroughly. Samples were incubated at 37°C, with shaking at 100 rpm, for 2 days. To investigate the effect of salt in soybean cultures, NaCl was added to soybean suspension to 7% (w/w). The samples were incubated at 37°C, with shaking at 100 rpm, for 10 days. After fermentation, supernatants were retrieved by centrifugation and stored at −70°C until analyses of FAAs.

### Analysis of FAAs

Samples (1 ml) were mixed with 50 ml of buffer containing 0.1 M perchloric acid and 0.1% metaphosphoric acid in distilled water. The mixture was sonicated for 1 h, shaken at room temperature for 1 h, and then filtered through a 0.2-μm membrane filter (Phenomenex, USA). The filtrate was analyzed by a custom service provided by the National Instrumentation Center for Environmental Management in Korea (http://nicem.snu.ac.kr/). The analyses were performed using an UltiMate 3000 HPLC system (Thermo Fisher Scientific, USA). Chromatographic separation was achieved with an Inno C18 column (4.6 × 150 mm, 5 μm; Young Jin Biochrom, Republic of Korea). Elution was carried out with 40 mM sodium phosphate buffer (solvent A, pH 7) and deionized water/acetonitrile/methanol (10:45:45 v/v/v; solvent B). The following binary mobile phase profile was used with linear gradients: 95% A at 0 min, 95% A at 3 min, 45% A at 24 min, 20% A at 56 min, and 95% A at 35 min. The column temperature and flow rate were 40°C and 1.5 ml/min, respectively. Detection was performed using a fluorescence detector. Three derivatizing agents, borate buffer (Agilent Technologies, USA), *o*-phthaldialdehyde (OPA; Agilent Technologies) and 9-fluorenylmethoxycarbonyl chloride (FMOC; Agilent Technologies), were used simultaneously according to the manufacturer’s instructions. The excitation/emission wavelengths were, respectively, 340/ 450 nm for OPA-derivatized amino acids and 266/305 nm for FMOC-derivatized amino acids. Data analysis was conducted using Chromeleon 6.8 software. Amino acid standards Agilent 5061-3330 and Agilent 5062-2478 (Agilent Technologies) were prepared at 1 nmol/μl in 0.1 M HCl and stored at −70°C. Quantification of each amino acid was performed by comparing with the peak areas of standard substances at four concentrations ranging from 10 to 1,000 pmol/μl. The correlation coefficient for all curves was > 0.99.

### Average nucleotide identity (ANI) Calculation

ANI was calculated using PYANI [12] for published complete genomes with BLASTN+ [13]. The published complete genome sequences for analysis were retrieved from the NCBI database.

### Statistical Analysis

All values were from independent triplicates and are expressed as the mean ± SD. Data were analyzed by one-way analysis of variance with Duncan’s test, performed using SPSS software v20.0 (IBM Corporation, USA). *P* < 0.05 indicated a statistically significant difference. To visualize the differences between the amino acids produced from sterilized soybeans by inoculated bacteria, principal component analysis (PCA) was applied with maximum variation rotation. The amino acid production was also visualized via heatmaps generated using the pheatmap package (v1.0.12). To determine the association between PI values and amino acid production, Pearson correlation analysis was performed using Python (v3.8.2) with Pandas for data handling, SciPy for statistical tests, and Matplotlib and Seaborn for visualization. The normality of the PI and amino acid data was tested using the Shapiro–Wilk test.

## Results

### Effects of NaCl on the Growth and Proteolytic Activity of Three *Bacillus* spp.

We tested the growth and proteolytic activity of 120 strains of each of *B. subtilis*, *B. velezensis*, and *B. licheniformis* in the presence of NaCl (Table 1). None of the strains could grow at an NaCl concentration of 15% (w/v). The proportion of strains that exhibited growth at 14% NaCl depended on the *Bacillus* species. Among the 120 *B. subtilis* strains, 54 (45%), 35 (29.2%), and 8 (6.7%) strains exhibited NaCl tolerances of 12%, 13%, and 14%, respectively. In the cases of *B. velezensis* and *B. licheniformis*, 87 (72.5%) and 92 (76.7%) strains showed growth at 14% NaCl, respectively. All the *B. velezensis* and *B. licheniformis* strains could grow on TSA in the presence of 12%NaCl, while all of the *B. subtilis* strains could grow in 10% NaCl.

The maximum NaCl concentrations that allowed proteolytic activity of *B. subtilis*, *B. velezensis*, and *B. licheniformis* were 13%, 13%, and <9%, respectively. Skim milk added to TSA decreased the water activity of the test agar plates and might have influenced the proteolytic activity in conditions where NaCl was added. All the *B. subtilis* and *B. velezensis* strains showed proteolytic activity at NaCl concentrations of 9% and 11%, respectively. Five *B. licheniformis* strains showed proteolytic activity only on the TSA containing 2% skim milk (*i.e.*, with no added NaCl).

Considering the growth and proteolytic activities of 120 strains of each of *B. subtilis*, *B. velezensis*, and *B. licheniformis* in the presence of NaCl, *B. subtilis* showed lower salt tolerance than *B. velezensis* and *B. licheniformis*. *B. velezensis* exhibited better growth than *B. subtilis* and higher proteolytic activity than *B. subtilis* and *B. licheniformis* in 13% NaCl. *B. licheniformis* showed the highest salt tolerance among the three species, but, in contrast to the other species, its proteolytic activity was not observed in high-salt conditions. Interestingly, strains with high NaCl tolerance did not necessarily show proteolytic activity at high NaCl concentrations. For example, the 92 *B. licheniformis* strains that were tolerant to 14% NaCl exhibited proteolytic activity at diverse maximum concentrations of NaCl.

### Salt Tolerance for Growth and Proteolytic Activity of Three *Bacillus* spp.

Among the test strains, 54 *B. subtilis* strains (45%) exhibited growth in 12% NaCl. Most (42, 77.8%) *B. subtilis* strains exhibiting growth at 12% NaCl showed proteolytic activity at 10% or 11% NaCl. Most (85, 70.8%) *B. velezensis* strains exhibited growth in 14% NaCl and showed proteolytic activity at 12% or 13% NaCl. Most *B. licheniformis* strains (68, 56.7%) exhibited growth in 14% NaCl and showed proteolytic activity at 5%–7% NaCl (Table 1).

**Fig. 1:**
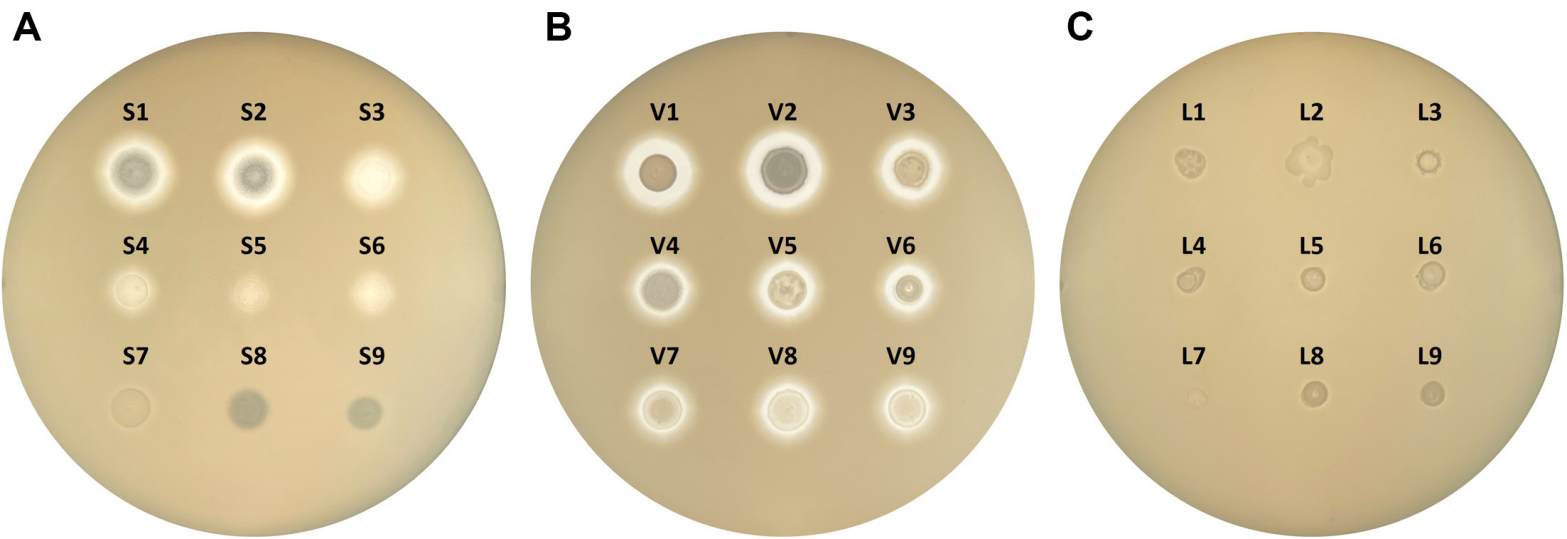
Growth and proteolytic activities of nine representative strains of each of *Bacillus subtilis* (A) *B. velezensis* (B) and *B. licheniformis* (C) on tryptic soy-agar plates containing 2% skim milk. *B. subtilis* strain abbreviations: S1, CH3-25; S2, F4241; S3, F1387; S4, SRCM103637; S5, F1237-2; S6, D12-5; S7, CHKJ 1253045; S8, F1323-1; and S9, PJ-7. *B. velezensis* strain abbreviations: V1, F2204; V2, GWHC B6; V3, MJ5-41; V4, SBK B3; V5, HCD2; V6, F1174-2; V7, Ms005; V8, 7BDL15; and V9, Rs501. *B. licheniformis* strain abbreviations: L1, SRCM100040; L2, F4112; L3, F3099-1; L4, 7DA21; L5, 7DA6; L6, 14DA18; L7, 8MS01; L8, 8MI08; and L9, SRCM100165.

We determined proteolytic activity semi-quantitatively to classify *Bacillus* strains having representative features of their species in terms of salt tolerance for growth and proteolytic activity. We selected nine strains of each of the three *Bacillus* spp. based on PI values (Fig. 1, Table 2). We classified the selected *B. subtilis* and *B. velezensis* strains as “high,” “medium,” and “low,” based on their activity levels. However, an activity difference was not clearly identified among the *B. licheniformis* strains. In general, *B. licheniformis* exhibits slower growth and lower proteolytic activity than *B. subtilis* and *B. velezensis*. Among the three species, *B. velezensis* exhibited the highest growth and proteolytic activity.

### FAA Profiles of *Bacillus*-Inoculated Soybean Cultures

**Fig. 2:**
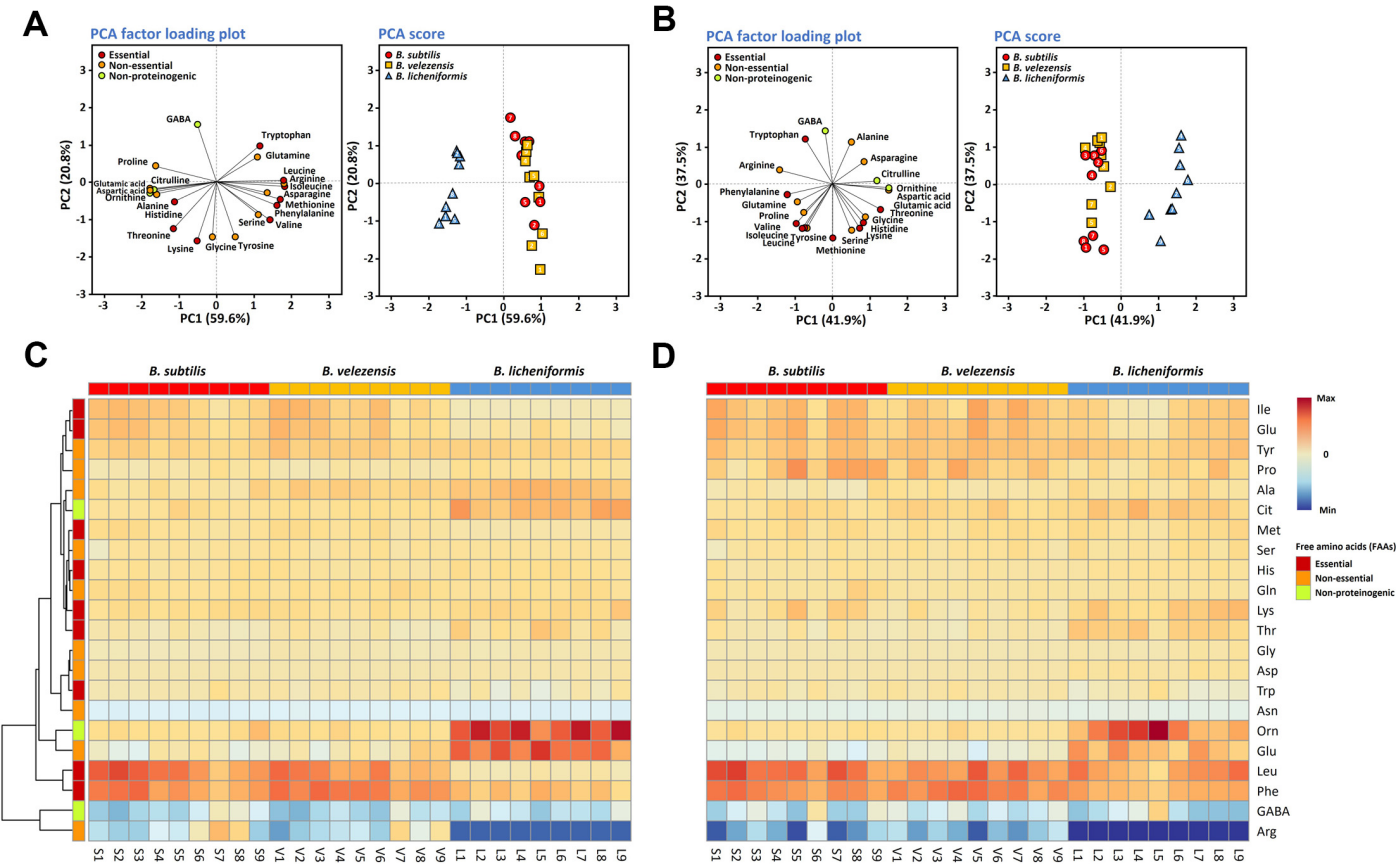
Principal component analysis (PCA) plots (A, B) and heatmaps (C, D) of the free amino acid (FAA) profiles of soybean cultures (A, C) and soybean cultures supplemented with 7% NaCl (B, D). *B. subtilis* strain abbreviations: 1 and S1, CH3-25; 2 and S2, F4241; 3 and S3, F1387; 4 and S4, SRCM103637; 5 and S5, F1237-2; 6 and S6, D12-5; 7 and S7, CHKJ 1253045; 8 and S8, F1323-1; and 9 and S9, PJ-7. *B. velezensis* strain abbreviations: 1 and V1, F2204; 2 and V2, GWHC B6; 3 and V3, MJ5-41; 4 and V4, SBK B3; 5 and V5, HCD2; 6 and V6, F1174-2; 7 and V7, Ms005; 8 and V8, 7BDL15; and 9 and V9, Rs501. *B. licheniformis* strain abbreviations: 1 and L1, SRCM100040; 2 and L2, F4112; 3 and L3, F3099-1; 4 and L4, 7DA21; 5 and L5, 7DA6; 6 and L6, 14DA18; 7 and L7, 8MS01; 8 and L8, 8MI08; and 9 and L9, SRCM100165.

Nine representative strains of each of the three *Bacillus* spp. were inoculated into sterile ground soybean suspensions and the FAAs in the cultures were analyzed. Twenty-two FAAs were quantified in the cultures, including the non-proteinogenic amino acids γ-aminobutyric acid, citrulline, and ornithine. The control samples were non-inoculated sterile ground soybean suspension. To minimize the errors occurring during soybean preparation processes, the quantities of FAAs in *Bacillus*-inoculated soybean cultures (Table S1) and *Bacillus*-inoculated soybean cultures supplemented with 7% NaCl (Table S2) were expressed as relative amino acid contents and then subjected to statistical analysis. Statistics on the 22 FAAs in the cultures normalized by the growth of the representative strains were subjected to PCA and heatmap analysis to represent the differences between samples in simple graphical ways (Fig. 2).

PCA score plots of FAA analysis data distinguished the FAA production by different *Bacillus* strains in soybean cultures. The strains of each species clustered together in PCA (Fig. 2A). The *B. licheniformis* cluster was separated from the clusters for *B. subtilis* and *B. velezensis*; the latter clusters overlapped. This indicates that the FAA production profile of *B. subtilis* in soybean cultures may be similar to that of *B. velezensis*. The clustering of each species in PCA was maintained in 7% NaCl-added cultures; and the similarity of the FAA profiles between *B. subtilis* and *B. velezensis* and the difference of the FAA profile of *B. licheniformis* compared with those of the other species were also maintained (Fig. 2B).

Heatmaps were drawn to represent the relative abundance of each FAA in the cultures. The similarity of FAA production profiles between *B. subtilis* and *B. velezensis* and the distinct FAA production profile of *B. licheniformis* observed in PCA were also identified in the heatmap analysis (Fig. 2C). The FAA production profiles of each *Bacillus* species in soybean cultures in the absence of added NaCl were similar to those in the presence of 7% NaCl (Fig. 2C and 2D). Considering the culture time of the NaCl-added soybean cultures, salt in the soybean cultures contributed to a decreased cell growth rate and proteolytic activity, but the FAA production profiles of each species in the soybean cultures were maintained even in the presence of the salt. On the basis of the clustering of PCA score plots and the similarities in heatmap analysis results within each species, the amino acid production profiles of *Bacillus* species can be considered characteristic of each species, rather than strain-specific.

We examined FAA production in soybean cultures at the species level, comparing *Bacillus*-inoculated cultures with the non-inoculated controls. When more than seven of the nine tested strains of a species had the same effect on an FAA, the change was considered statistically significant (Table S1). The contents of asparagine, aspartic acid, glycine, and serine were all significantly decreased in the cultures of all three *Bacillus* spp. *B. subtilis* increased the amounts of leucine and phenylalanine. *B. velezensis* increased the amounts of leucine, phenylalanine, and tyrosine. *B. licheniformis* increased the amounts of alanine, glutamic acid, tyrosine, and ornithine, and dramatically decreased the content of arginine. The decrease of asparagine, glycine, and serine by all three *Bacillus* spp. was also identified in the cultures containing 7% NaCl (Table S2). In NaCl-supplemented cultures, the *B. subtilis*-specific increases of leucine and phenylalanine and the *B. velezensis*-specific increase of phenylalanine were maintained. No *B. licheniformis*-specific increase in amino acid contents was identified in the presence of 7% NaCl, but the dramatic decrease in arginine content was maintained. The marked decrease in the proteolytic activity of *B. licheniformis* in the presence of 7% NaCl might delay any species-specific amino acid production.

### Correlations between PI Values and Amino Acid Production

**Fig. 3:**
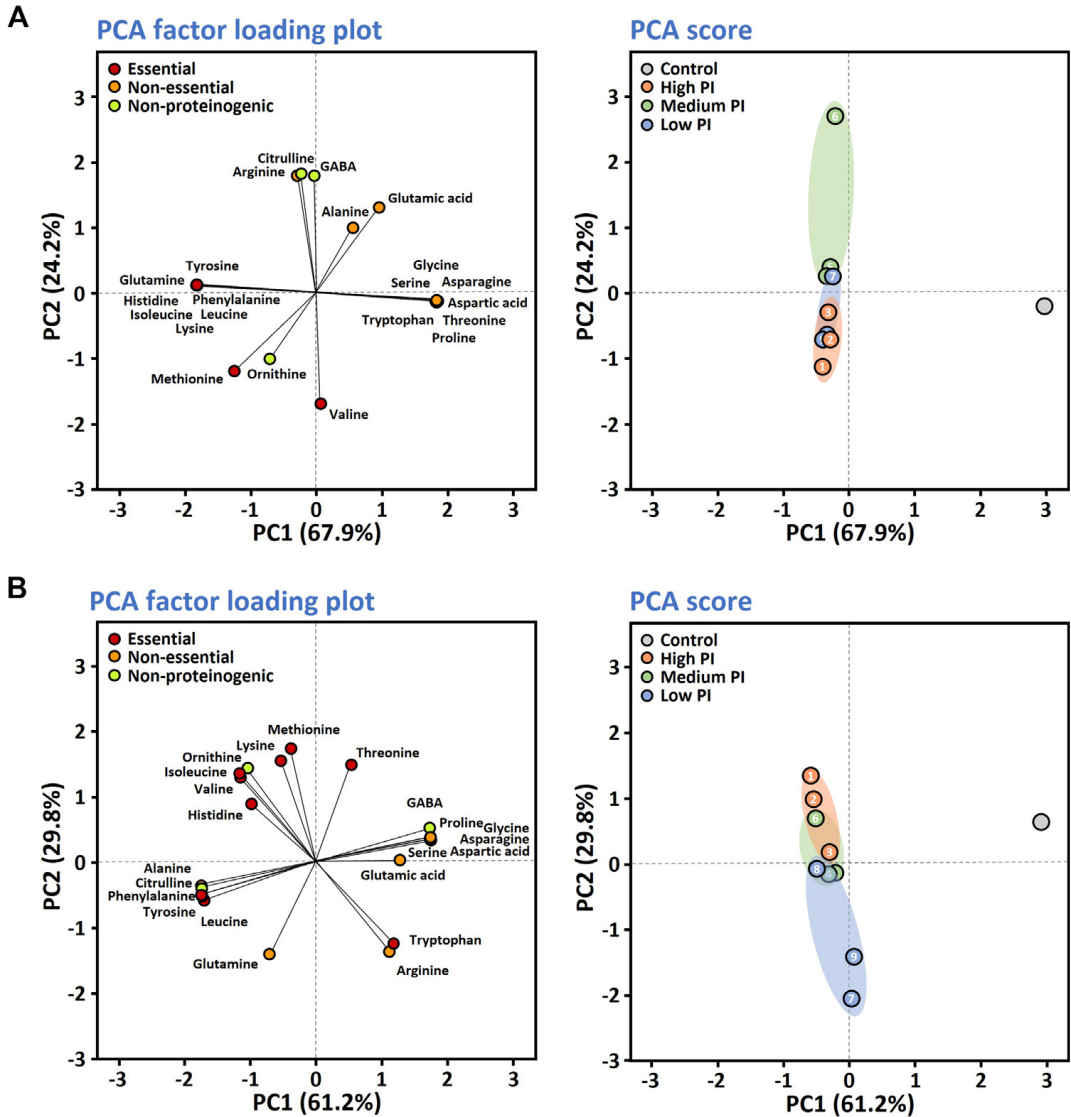
PCA results for the FAA profiles from soybean cultures of *B. subtilis* (A) and *B. velezensis* (B) analyzed at the species level. *B. subtilis* strain abbreviations: 1, CH3-25; 2, F4241; 3, F1387; 4, SRCM103637; 5, F1237-2; 6, D12-5; 7, CHKJ 1253045; 8, F1323-1; and 9, PJ-7. *B. velezensis* strain abbreviations: 1, F2204; 2, GWHC B6; 3, MJ5-41; 4, SBK B3; 5, HCD2; 6, F1174-2; 7, Ms005; 8, 7BDL15; and 9, Rs501. The analysis results for sterilized soybean samples were used as the control. Semi-quantitative determination of proteolytic activity used the proteolytic index (PI) value and *Bacillus* strains were classified as “high,” “medium,” and “low,” based on their PI values.

When the FAA profiles were analyzed at the species level, the PCA score plots for *B. subtilis* and *B. velezensis* tended to cluster according to PI values (Fig. 3). *B. licheniformis* strains did not cluster according to PI values. The PCA score plots of *B. subtilis* strains showing high PI values were influenced by the contents of methionine, ornithine, and valine. The contents of methionine, lysine, ornithine, isoleucine, valine, and histidine affected the PCA score plots of *B. velezensis* strains having high PI values.

To statistically test correlations between PI values and amino acid production by *B. subtilis* and *B. velezensis*, the Pearson correlation test, which measures the strength and direction of a linear relationship between two continuous variables, was applied. The Pearson correlation coefficient (PCC) quantifies this linear relationship with a value ranging from −1 to 1 (Table 3, Fig. S1). The PI values of *B. subtilis* strains showed moderate positive correlations with the amounts of methionine and valine and moderate negative correlation with that of ornithine. The PI values of *B. velezensis* strains had strong positive correlations with methionine, isoleucine, and valine. The PI values of *B. velezensis* exhibited moderate correlations with lysine, ornithine, and histidine. Among the amino acids that were specifically produced by *B. subtilis* in this study, leucine had a moderate positive correlation with PI values, but phenylalanine had a weak correlation. The PI values for *B. velezensis* had strong positive correlations with the amount of leucine and moderate positive correlations with phenylalanine and tyrosine. Among the 22 FAAs quantified in this study, the amounts of seven FAAs produced by *B. velezensis* had strong correlations with PI values and 13 had moderate correlations. Thus, in the case of *B. velezensis*, Pearson correlation tests show that the quantities of amino acids produced in soybean cultures depend on the proteolytic activity of the strain. The PI value may be applied for determination of the proteolytic activity of *B. velezensis*, but not *B. subtilis*. The morphological characteristics of *B. velezensis*, which form distinctive colonies and clear proteolytic zones, might contribute to the reliable determination of PI values for that species (Fig. 1).

## Discussion

Predominance of *Bacillus* spp. in fermented soybean foods has been reported not only in culture-dependent microbial community analyses in past decades but also in the “omics” studies of recent years [14, 15]. The contribution of *Bacillus* spp. to fast-fermented soybean food products, including *cheonggukjang* (from Korea) and *natto* (Japan), was well explained in studies illuminating their prevalence and metabolite production [16-18]. However, their roles in high-salt fermented soybean foods such as *doenjang* (Korea) and *dajiang* (China) remain in question because *Bacillus* form endospores in high-salt conditions. In a microbial community migration study of *doenjang*, a traditional Korean fermented soybean paste that is prepared by mixing *meju* (fermented soybean block) with solar salt brine (to approximately 18% NaCl), the spore formation and inactiveness of *Bacillus* during the fermentation were noted [19]. However, on the basis of its high salt tolerance and detection number in high-salt conditions, we considered *B. licheniformis* as a functional starter culture candidate for high-salt soybean fermentations [20]. In the present study, we confirmed the growth of *B. velezensis* and *B. licheniformis* at the NaCl concentration of 14% (w/v) and a dramatic decrease in the proteolytic activity of *B. licheniformis* at NaCl concentration >7%. Considering its salt tolerance and proteolytic activity, *B. velezensis* might have greater potential than *B. licheniformis* to contribute to the ripening of high-salt fermented soybean foods.

Rapid progress in molecular taxonomic methodologies and introduction of phylogenomic approaches have led to taxonomic changes in bacteria including *Bacillus* species. *B. velezensis* was isolated and first designated by Ruiz-Garcia *et al*. [21]. Later phylogenomic analysis reclassified *B. amyloliquefaciens* subsp. *plantarum*, *B. methylotrophicus*, and *B. oryzicola* as *B. velezensis* [22]. Since the reestablishment of its taxonomic status, *B. velezensis* has been the species identified most often in fermented soybean foods [23-25]. The high recovery of *B. velezensis* in recent studies of fermented soybean foods might be associated with its high salt tolerance and high proteolytic activity. Considering its prevalence, salt tolerance, and proteolytic activity, *B. velezensis* can be a potential starter candidate for fermented soybean foods, even those produced in high-salt conditions.

Strain specificity in bacteria refers to the unique characteristics and behaviors exhibited by different strains of the same bacterial species. Strain specificity highlights the diversity and unique features of bacterial strains and explains their behavior, metabolism, and ecological roles in a niche. Strain specificity of bacteria has been underscored in studies of probiotic efficacy, pathogenicity, and antibiotic resistance [26, 27]. Amino acids serve as a source of energy and constituent materials for cells. Proteases of chemoheterotrophic bacteria help use protein sources, providing amino acids for their survival. In this study, the similarities of FAA profiles from soybean cultures for nine representative strains of each of three *Bacillus* spp. led to the tentative conclusion that the amino acid production profile is a characteristic of the species, not the strains.

*B. subtilis*, the type species of the genus *Bacillus*, has been a representative research target for the food fermentation industry because of its prevalence in foods, proven safety, long history of use, and probiotic activity [28]. Several studies have been performed that illuminate the metabolic traits of *B. subtilis* strains in soybean-based fermented foods and mentioned an increase in the essential amino acids in the food matrix. Gao *et al*. [29] reported an increase of lysine, isoleucine, valine, leucine, phenylalanine, and tyrosine in soymilk on fermentation by *B. subtilis*. A metabolomic study to understand the metabolites from the fermentation of *okara*, a major agrowaste produced from soymilk and bean curd producing processes, reported increased leucine, phenylalanine, and glutamic acid [30]. In our previous study to evaluate the amino acid profiles of *B. subtilis*-fermented beans, an increase in phenylalanine, leucine, and valine was identified from soybean, chickpea, and fava bean [31]. Our previous comparison of FAA production in soybean fermentations using four strains of four *Bacillus* spp. mentioned the similarity of the FAA production profiles between *B. subtilis* and *B. velezensis* compared with those of *B. licheniformis* and *B. sonorensis* [32]. All of these studies support the present result that *B. subtilis* specifically increases leucine and phenylalanine through soybean fermentation, and the similarity of amino acid production profiles between *B. subtilis* and *B. velezensis*. The study of Seo *et al*. [31] indicates that the amino acid production is independent of the food matrix.

In our previous study, we selected five genome-published *B. subtilis* strains showing different salt tolerances and proteolytic activities and identified potential candidate determinants of phenotypic differences via comparative genomic analysis [33]. We gained the insight that possession of additional gene homologs for the *B. subtilis* proteolytic system might contribute to differences in the proteolytic activities of different strains. Through the present study, we determined that the amino acid profiles produced by *B. subtilis* and *B. licheniformis* strains might not be determined by their proteolytic activity. However, in the case of *B. velezensis*, the strength of proteolytic activity might influence quantitative differences in amino acids produced in fermentations. Our results regarding the production of amino acids at the species level and the correlations between proteolytic activity and amino acid quantity will facilitate the determination and selection of target strains for functional *Bacillus*-fermented foods.

Among the complete genome-published *Bacillus* strains, the genomic sequences of 13 *B. subtilis* strains, 15 *B. velezensis* strains, and 8 *B. licheniformis* strains, identified from fermented soybean foods, were retrieved for ANI analysis. *B. subtilis* strains exhibited 77.56%–78.04% identity with *B. velezensis* strains, and 74.36%–74.94%identity with *B. licheniformis* strains (Fig. S2). The ANI between *B. velezensis* and *B. licheniformis* was 74.22%–74.58%. These analyses indicate the relative genetic closeness between *B. subtilis* and *B. velezensis* compared with *B. licheniformis*. That closeness can be linked to the similarity of amino acid metabolism between *B. subtilis* and *B. velezensis*.

## Supplemental Materials

Supplementary data for this paper are available on-line only at http://jmb.or.kr.



## Figures and Tables

**Table 1:** Numbers of *Bacillus* strains that showed growth and proteolytic activity on tryptic soy-agar (TSA) plates containing NaCl.

Species	NaCl tolerance for proteolytic activity (% w/v)^1)^	NaCl tolerance for growth (% w/v)					Total
		10	11	12	13	14	
*B. subtilis*	9	2	1	–	1	–	4
	10	4	11	20	15	3	53
	11	–	5	22	8	2	37
	12	–	–	12	10	1	23
	13	–	–	–	1	2	3
*B. velezensis*	9	–	–	–	–	–	–
	10	–	–	–	–	–	–
	11	–	–	–	7	2	9
	12	–	–	7	13	42	62
	13	–	–	–	6	43	49
*B. licheniformis*	0.5	–	–	–	–	5	5
	1	–	–	–	–	5	5
	3	–	–	–	4	14	18
	5	–	–	2	12	40	54
	7	–	–	3	7	28	38
	9	–	–	–	–	–	–

^1)^Proteolytic activity was determined on TSA supplemented with 2% (w/v) skim milk and NaCl was added to determine the maximum NaCl concentration that allowed proteolytic activity.

**Table 2:** Proteolytic index (PI) values of nine representative strains of each of three *Bacillus* spp.

Species	PI value classification	Strain abbreviation	Strain	PI value
*B. subtilis*	High	S1	CH3-25	1.46 ± 0.02
		S2	F4241	1.44 ± 0.02
		S3	F1387	1.43 ± 0.02
	Medium	S4	SRCM103637	1.28 ± 0.02
		S5	F1237-2	1.25 ± 0.05
		S6	D12-5	1.25 ± 0.03
	Low	S7	CHKJ 1253045	1.07 ± 0.01
		S8	F1323-1	0.00 ± 0.00
		S9	PJ-7	0.00 ± 0.00
*B. velezensis*	High	V1	F2204	1.91 ± 0.03
		V2	GWHC B6	1.88 ± 0.03
		V3	MJ5-41	1.84 ± 0.04
	Medium	V4	SBK B3	1.59 ± 0.04
		V5	HCD2	1.55 ± 0.02
		V6	F1174-2	1.54 ± 0.02
	Low	V7	Ms005	1.35 ± 0.01
		V8	7BDL15	1.30 ± 0.00
		V9	Rs501	1.30 ± 0.03
*B. licheniformis*	1)	L1	SRCM100040	1.17 ± 0.04
		L2	F4112	1.17 ± 0.05
		L3	F3099-1	1.15 ± 0.00
		L4	7DA21	1.15 ± 0.03
		L5	7DA6	1.14 ± 0.05
		L6	14DA18	1.10 ± 0.01
		L7	8MS01	1.09 ± 0.01
		L8	8MI08	1.09 ± 0.00
		L9	SRCM100165	1.07 ± 0.01

^1)^Classification of proteolytic activity of *B. licheniformis* strains was not applicable based on the PI values.

**Table 3:** Pearson correlation coefficients (PCCs) between PI values of, and free amino acid production by, *B. subtilis* and *B. velezensis*.

Amino acid		*B. subtilis*		*B. velezensis*	
		PCC value (r)	Correlation strength	PCC value (r)	Correlation strength
Essential	Histidine	−0.64	Moderate	0.58	Moderate
	Isoleucine	0.52	Moderate	0.87**	Strong
	Leucine	0.56	Moderate	0.88**	Strong
	Lysine	−0.59	Moderate	0.56	Moderate
	Methionine	0.53	Moderate	0.80**	Strong
	Phenylalanine	0.44	Weak	0.73*	Moderate
	Threonine	0.62	Moderate	0.85**	Strong
	Tryptophan	−0.31	Weak	−0.59	Moderate
	Valine	0.52	Moderate	0.91**	Strong
Non-essential	Alanine	−0.30	Weak	0.18	Negligible
	Arginine	−0.21	Negligible	−0.87**	Strong
	Asparagine	−0.59	Moderate	0.41	Weak
	Aspartic acid	0.02	Negligible	−0.11	Negligible
	Glutamic acid	0.27	Weak	−0.72*	Moderate
	Glutamine	0.30	Weak	−0.59	Moderate
	Glycine	0.68*	Moderate	0.88**	Strong
	Proline	−0.58	Moderate	−0.64	Moderate
	Serine	0.60	Moderate	0.52	Moderate
	Tyrosine	0.27	Weak	0.64	Moderate
Non-proteinogenic	Citrulline	0.16	Negligible	−0.57	Moderate
	γ-Aminobutyric acid	−0.67	Moderate	−0.73**	Moderate
	Ornithine	−0.70*	Moderate	0.52	Moderate

Correlation strength was determined according to absolute PCC value: 0 < r < 0.25, negligible; 0.25 < r < 0.5, weak; 0.5 < r < 0.75, moderate; 0.75 < r < 1, strong.

*Values considered significant (*p* < 0.05). **Values considered highly significant (*p* < 0.01).
