# Supplementary material for: Specificity of Amino Acid Profiles Produced in Soybean Fermentations by Three Bacillus spp
Source: J Microbiol Biotechnol. 2024 Dec 2;35:e2411038. doi: 10.4014/jmb.2411.11038 (PMC11813359; doi:10.4014/jmb.2411.11038)

J. Microbiol. Biotechnol. 2025. 35: e2411038

https://doi.org/10.4014/jmb.2411.11038

Supplementary Tables and Figures

Table S1. Percentage concentrations of free amino acids in *Bacillus* strain-inoculated soybean cultures.

Species	Strain	Essential amino acid									Non-essential amino acid										Non-proteinogenic amino acid		
		His	Ile	Leu	Lys	Met	Phe	Thr	Trp	Val	Ala	Arg	Asn	Asp	Glu	Gln	Gly	Pro	Ser	Tyr	Cit	GABA	Orn
–	Control	2.04^{ab}	2.05^{ghij}	2.85^{f}	2.70^{b}	1.13^{cdefgh}	3.33^{l}	2.42^{cdef}	5.02^{abc}	2.63^{ghijk}	2.47^{jkl}	26.38^{abc}	6.43^{a}	1.44^{a}	6.57^{fghi}	0.20^{c}	1.98^{a}	2.62^{abc}	2.81^{a}	2.75^{f}	0.23^{d}	21.92^{a}	0.00^{d}
B. subtilis	S1	1.87^{ab}	6.79^{abcd}	17.78^{ab}	2.93^{b}	2.07^{abc}	15.06^{abcde}	1.39^{def}	3.49^{abcd}	6.77^{abcdef}	3.33^{ijkl}	16.41^{de}	0.48^{b}	0.20^{fg}	4.17^{ghi}	1.30^{abc}	0.21^{defg}	1.17^{c}	0.28^{cd}	5.13^{bcde}	0.29^{d}	8.15^{cde}	0.74^{d}
	S2	2.39^{ab}	7.56^{ab}	19.85^{a}	3.91^{b}	2.75^{a}	16.15^{ab}	1.74^{def}	3.51^{abcd}	7.88^{ab}	2.07^{kl}	13.06^{ef}	0.33^{b}	0.14^{fg}	2.28^{hi}	1.36^{abc}	0.21^{defg}	1.70^{abc}	0.37^{cd}	6.06^{abc}	0.26^{d}	5.07^{e}	1.37^{d}
	S3	2.64^{ab}	6.56^{abcd}	17.53^{ab}	4.00^{b}	2.29^{ab}	17.16^{a}	1.17^{ef}	3.60^{abcd}	7.44^{abc}	2.39^{jkl}	13.01^{ef}	0.43^{b}	0.28^{defg}	1.44^{i}	1.75^{bc}	0.13^{defg}	1.17^{c}	0.48^{bcd}	5.84^{bcd}	0.23^{d}	9.78^{bcde}	0.68^{d}
	S4	1.66^{ab}	5.96^{abcde}	15.40^{abc}	3.22^{b}	1.42^{bcdefg}	11.18^{cdefghi}	0.56^{ef}	3.97^{abc}	5.37^{bcdefg}	3.75^{hijkl}	20.37^{bcde}	0.58^{b}	0.21^{fg}	6.23^{fghi}	0.65^{bc}	0.16^{defg}	2.00^{abc}	0.29^{cd}	4.26^{def}	0.56^{cd}	11.45^{abcde}	0.75^{d}
	S5	2.14^{ab}	5.42^{abcdef}	15.53^{abc}	3.76^{b}	1.53^{bcdef}	13.03^{abcdefgh}	2.01^{def}	3.68^{abc}	5.47^{bcdefg}	2.17^{kl}	18.74^{bcde}	0.39^{b}	0.27^{defg}	5.50^{fghi}	0.53^{bc}	0.20^{defg}	1.82^{abc}	0.40^{cd}	5.08^{bcde}	2.62^{abcd}	9.11^{bcde}	0.59^{d}
	S6	1.89^{ab}	3.73^{defgh}	11.90^{bcde}	3.47^{b}	1.08^{cdefgh}	10.58^{defghij}	0.91^{ef}	3.66^{abc}	4.47^{defghi}	2.48^{jkl}	24.69^{abcd}	0.37^{b}	0.20^{fg}	6.71^{fgh}	0.51^{bc}	0.11^{fg}	2.26^{abc}	0.14^{d}	4.35^{cdef}	1.37^{bcd}	14.76^{abcde}	0.36^{d}
	S7	2.20^{ab}	1.56^{hij}	6.26^{ef}	4.12^{b}	0.80^{defgh}	6.96^{ijkl}	0.53^{ef}	5.79^{a}	3.17^{ghijk}	2.19^{kl}	31.90^{a}	0.28^{b}	0.25^{efg}	4.96^{fghi}	0.49^{bc}	0.02^{g}	3.28^{a}	0.22^{cd}	3.87^{ef}	0.78^{bcd}	20.13^{ab}	0.25^{d}
	S8	2.78^{ab}	3.36^{efghi}	10.06^{cde}	4.08^{b}	0.90^{defgh}	9.95^{fghij}	0.31^{ef}	4.14^{abc}	3.71^{ghijk}	1.70^{l}	28.21^{ab}	0.46^{b}	0.26^{efg}	2.14^{hi}	0.94^{abc}	0.01^{g}	2.26^{abc}	0.17^{d}	4.20^{def}	0.33^{d}	18.71^{abc}	1.35^{d}
	S9	2.71^{ab}	4.29^{cdefgh}	12.14^{bcde}	4.33^{b}	1.41^{bcdefg}	12.02^{bcdefgh}	0.61^{ef}	4.24^{abc}	5.29^{bcdefgh}	5.18^{defghi}	12.74^{ef}	0.85^{b}	0.16^{fg}	3.43^{hi}	0.68^{bc}	0.09^{fg}	2.92^{abc}	0.17^{d}	5.31^{bcde}	0.49^{d}	15.82^{abcde}	5.12^{d}
B. velezensis	V1	2.83^{ab}	8.08^{a}	16.56^{ab}	3.86^{b}	2.38^{ab}	16.68^{ab}	1.77^{def}	4.54^{abc}	8.45^{a}	3.70^{hijkl}	6.66^{fg}	0.65^{b}	0.26^{efg}	4.08^{ghi}	0.38^{c}	0.94^{b}	1.26^{bc}	0.74^{bcd}	7.46^{a}	0.72^{bcd}	6.57^{e}	1.45^{d}
	V2	1.84^{ab}	8.25^{a}	15.10^{abc}	3.65^{b}	2.39^{ab}	13.88^{abcdef}	2.45^{cdef}	3.14^{abcde}	7.29^{abcd}	6.19^{bcdefg}	12.37^{ef}	1.27^{b}	0.24^{fg}	5.66^{fghi}	1.05^{abc}	0.94^{b}	1.23^{c}	1.10^{b}	5.21^{bcde}	0.92^{bcd}	4.83^{e}	1.01^{d}
	V3	1.97^{ab}	6.69^{abcd}	13.90^{abcd}	2.94^{b}	1.86^{abcd}	17.01^{a}	1.69^{def}	4.12^{abc}	7.16^{abcde}	4.77^{efghi}	15.21^{def}	0.94^{b}	0.11^{g}	2.20^{hi}	0.59^{bc}	0.52^{bcdefg}	1.19^{c}	0.21^{cd}	5.70^{bcd}	0.38^{d}	10.00^{bcde}	0.83^{d}
	V4	1.70^{ab}	4.06^{cdefgh}	9.94^{cde}	2.69^{b}	1.16^{cdefgh}	15.56^{abc}	0.84^{ef}	4.55^{abc}	4.90^{cdefgh}	5.59^{cdefgh}	17.32^{cde}	0.69^{b}	0.26^{efg}	5.12^{fghi}	1.04^{abc}	0.46^{bcdefg}	2.21^{abc}	0.21^{cd}	5.63^{bcd}	0.69^{bcd}	13.27^{abcde}	2.10^{d}
	V5	1.75^{ab}	4.92^{bcdefg}	10.38^{cde}	2.72^{b}	1.62^{bcde}	15.38^{abcd}	1.09^{ef}	4.06^{abc}	5.24^{bcdefgh}	5.34^{cdefghi}	18.68^{bcde}	0.76^{b}	0.27^{defg}	6.89^{fgh}	0.79^{abc}	0.72^{bcd}	2.06^{abc}	0.37^{cd}	5.79^{bcd}	0.90^{bcd}	9.42^{bcde}	0.86^{d}
	V6	2.24^{ab}	7.11^{abc}	14.74^{abcd}	3.72^{b}	2.29^{ab}	14.97^{abcde}	1.98^{def}	3.90^{abc}	7.06^{abcde}	4.13^{ghijk}	13.18^{ef}	0.97^{b}	0.20^{fg}	4.16^{ghi}	0.82^{abc}	0.57^{bcdefg}	1.64^{abc}	0.91^{bc}	6.29^{ab}	0.94^{bcd}	7.35^{de}	0.84^{d}
	V7	1.88^{ab}	2.17^{ghij}	6.93^{ef}	3.25^{b}	0.53^{efgh}	10.47^{efghij}	0.18^{f}	4.92^{abc}	3.11^{ghijk}	4.22^{fghijk}	28.15^{ab}	0.76^{b}	0.17^{fg}	5.26^{fghi}	1.98^{a}	0.25^{cdefg}	2.27^{abc}	0.10^{d}	4.09^{def}	0.74^{bcd}	18.21^{abcd}	0.34^{d}
	V8	1.53^{b}	4.15^{cdefgh}	8.85^{de}	2.45^{b}	1.31^{bcdefgh}	13.26^{abcdefg}	0.44^{ef}	4.36^{abc}	4.14^{fghij}	4.86^{efghi}	21.98^{bcde}	0.75^{b}	0.21^{fg}	9.46^{ef}	1.26^{abc}	0.19^{defg}	1.67^{abc}	0.38^{cd}	4.96^{bcde}	1.07^{bcd}	12.03^{abcde}	0.70^{d}
	V9	1.79^{ab}	2.57^{fghij}	7.33^{ef}	2.91^{b}	0.79^{defgh}	12.50^{abcdefgh}	0.35^{ef}	5.22^{ab}	3.46^{ghijk}	4.42^{fghij}	26.20^{abc}	0.75^{b}	0.27^{defg}	9.21^{efg}	0.82^{abc}	0.12^{efg}	1.64^{abc}	0.27^{cd}	4.96^{bcde}	1.04^{bcd}	13.09^{abcde}	0.28^{d}
B. licheniformis	L1	2.97^{ab}	0.40^{ij}	2.01^{f}	3.97^{b}	0.31^{gh}	9.06^{fghijk}	5.38^{ab}	2.46^{cde}	1.51^{jk}	6.37^{bcdef}	0.20^{g}	0.27^{b}	0.44^{cdefg}	21.07^{abc}	0.39^{c}	0.65^{bcdef}	3.18^{a}	0.33^{cd}	5.24^{bcde}	9.51^{a}	8.79^{cde}	15.48^{b}
	L2	2.23^{ab}	0.37^{ij}	1.38^{f}	4.28^{b}	0.20^{h}	5.97^{jkl}	1.51^{def}	4.72^{abc}	1.76^{ijk}	5.62^{cdefgh}	0.12^{g}	0.24^{b}	0.62^{bcde}	16.68^{cd}	0.30^{c}	0.36^{bcdefg}	3.33^{a}	0.23^{cd}	5.37^{bcde}	4.53^{abcd}	18.33^{abcd}	21.87^{a}
	L3	2.80^{ab}	0.17^{j}	1.19^{f}	4.75^{ab}	0.19^{h}	9.08^{fghijk}	3.09^{bcde}	1.07^{de}	2.69^{ghijk}	7.26^{abcd}	0.17^{g}	0.24^{b}	0.45^{cdefg}	22.72^{ab}	0.38^{c}	0.34^{cdefg}	2.60^{abc}	0.30^{cd}	5.18^{bcde}	3.36^{abcd}	15.09^{abcde}	16.89^{b}
	L4	2.65^{ab}	0.29^{ij}	1.53^{f}	4.40^{b}	0.42^{fgh}	6.01^{jkl}	2.22^{def}	3.57^{abcd}	1.49^{jk}	8.56^{a}	0.18^{g}	0.39^{b}	0.49^{cdefg}	17.31^{cd}	0.95^{abc}	0.80^{bc}	2.93^{abc}	0.33^{cd}	4.48^{bcde}	5.83^{abcd}	13.32^{abcde}	21.84^{a}
	L5	2.84^{ab}	0.34^{ij}	2.23^{f}	3.60^{b}	0.34^{gh}	10.44^{efghij}	6.55^{a}	0.90^{e}	4.33^{efghij}	8.87^{a}	0.28^{g}	0.34^{b}	0.74^{bc}	25.55^{a}	0.71^{abc}	0.70^{bcde}	2.52^{abc}	0.28^{cd}	5.30^{bcde}	5.63^{abcd}	7.57^{de}	9.97^{c}
	L6	3.09^{a}	0.18^{j}	1.38^{f}	4.49^{b}	0.25^{h}	8.79^{ghijk}	4.89^{abc}	2.58^{bcde}	2.98^{ghijk}	7.80^{ab}	0.17^{g}	0.20^{b}	0.48^{cdefg}	19.20^{bc}	0.41^{c}	0.53^{bcdefg}	3.21^{a}	0.27^{cd}	5.08^{bcde}	6.14^{abcd}	12.76^{abcde}	15.12^{b}
	L7	2.61^{ab}	0.48^{ij}	1.95^{f}	4.08^{b}	0.39^{gh}	6.19^{jkl}	1.84^{def}	4.23^{abc}	1.15^{k}	7.45^{abc}	0.12^{g}	0.50^{b}	0.64^{bcd}	19.27^{bc}	1.33^{abc}	0.82^{bc}	2.68^{abc}	0.44^{cd}	4.57^{bcde}	4.26^{abcd}	12.44^{abcde}	22.55^{a}
	L8	2.67^{ab}	0.54^{ij}	2.31^{f}	3.80^{b}	0.48^{fgh}	8.22^{hijk}	4.04^{abcd}	2.93^{bcde}	2.47^{hijk}	6.89^{abcde}	0.26^{g}	0.54^{b}	0.51^{cdef}	20.57^{bc}	1.28^{abc}	0.80^{bc}	3.07^{ab}	0.43^{cd}	4.74^{bcde}	8.07^{abc}	10.63^{bcde}	14.78^{b}
	L9	2.04^{ab}	0.34^{ij}	1.26^{f}	6.61^{a}	0.23^{h}	4.40^{kl}	0.84^{ef}	4.83^{abc}	0.94^{k}	5.77^{bcdefgh}	0.14^{g}	0.28^{b}	0.88^{b}	13.46^{de}	0.24^{c}	0.67^{bcdef}	2.46^{abc}	0.28^{cd}	4.59^{bcde}	8.16^{ab}	17.79^{abcd}	23.77^{a}

Different superscripts within a column denote a significant difference between mean values ($p<0.05$) according to Duncan's multiple range test.

Table S2. Percentage concentrations of free amino acids in *Bacillus* strain-inoculated soybean cultures supplemented with 7% (w/v) NaCl.

Species	Strain	Essential amino acid								
		His	Ile	Leu	Lys	Met	Phe	Thr	Trp	Val
–	Control	2.47^{a}	2.22^{cde}	3.30^{e}	3.52^{bcdef}	1.06^{de}	3.29^{f}	2.47^{ef}	4.51^{ab}	2.92^{cd}
B. subtilis	S1	2.91^{a}	9.95^{ab}	19.80^{ab}	6.05^{abcdef}	3.02^{a}	15.75^{abc}	2.82^{cdef}	2.43^{bcd}	10.26^{a}
	S2	1.58^{a}	6.81^{abcde}	21.52^{a}	2.51^{def}	1.70^{abcde}	16.86^{a}	1.44^{ef}	3.44^{abcd}	7.61^{abcd}
	S3	1.77^{a}	4.86^{abcde}	15.56^{abcd}	2.87^{def}	1.39^{abcde}	14.63^{abcd}	1.10^{f}	3.85^{abc}	6.21^{abcd}
	S4	2.11^{a}	6.76^{abcde}	16.21^{abc}	4.69^{abcdef}	1.80^{abcde}	13.31^{abcd}	1.08^{f}	4.11^{ab}	8.14^{abc}
	S5	2.17^{a}	8.36^{abc}	16.82^{abc}	8.79^{ab}	2.90^{abc}	11.93^{abcde}	2.46^{ef}	2.80^{bcd}	9.60^{ab}
	S6	1.96^{a}	2.79^{cde}	11.35^{abcde}	3.20^{cdef}	0.99^{de}	9.64^{bcdef}	0.37^{f}	4.52^{ab}	4.31^{bcd}
	S7	2.68^{a}	8.08^{abcd}	19.13^{ab}	6.67^{abcdef}	2.37^{abcd}	13.89^{abcd}	2.62^{def}	2.99^{bcd}	8.83^{ab}
	S8	2.67^{a}	8.00^{abcd}	15.79^{abcd}	6.38^{abcdef}	2.49^{abcd}	13.43^{abcd}	1.26^{ef}	2.76^{bcd}	8.52^{ab}
	S9	2.09^{a}	3.55^{bcde}	10.23^{abcde}	2.96^{def}	1.23^{cde}	10.48^{abcde}	0.56^{f}	4.20^{ab}	5.16^{abcd}
B. velezensis	V1	1.80^{a}	3.45^{bcde}	10.00^{abcde}	2.31^{ef}	1.03^{de}	14.21^{abcd}	0.68^{f}	4.30^{ab}	5.02^{abcd}
	V2	2.09^{a}	6.53^{abcde}	13.56^{abcde}	5.05^{abcdef}	2.10^{abcde}	12.42^{abcde}	1.40^{ef}	3.27^{abcd}	7.64^{abcd}
	V3	1.87^{a}	4.74^{abcde}	12.26^{abcde}	2.30^{ef}	1.31^{bcde}	15.40^{abc}	0.87^{f}	4.04^{abc}	6.74^{abcd}
	V4	1.67^{a}	2.82^{cde}	11.28^{abcde}	1.60^{f}	0.80^{de}	17.12^{a}	0.30^{f}	4.53^{ab}	6.43^{abcd}
	V5	2.55^{a}	10.19^{a}	18.73^{abc}	3.69^{abcdef}	2.44^{abcd}	16.67^{ab}	2.37^{ef}	3.20^{abcd}	10.47^{a}
	V6	2.04^{a}	6.09^{abcde}	12.46^{abcde}	2.52^{def}	1.80^{abcde}	14.63^{abcd}	1.28^{ef}	3.79^{abc}	6.80^{abcd}
	V7	1.95^{a}	9.42^{ab}	17.54^{abc}	3.41^{cdef}	2.21^{abcd}	15.01^{abc}	1.92^{ef}	2.96^{bcd}	8.67^{ab}
	V8	1.87^{a}	6.35^{abcde}	13.30^{abcde}	4.04^{abcdef}	1.74^{abcde}	11.03^{abcde}	1.35^{ef}	3.47^{abcd}	6.47^{abcd}
	V9	1.89^{a}	4.29^{abcde}	10.92^{abcde}	2.50^{def}	1.30^{bcde}	13.82^{abcd}	0.91^{f}	4.02^{abc}	5.44^{abcd}
B. licheniformis	L1	1.90^{a}	5.45^{abcde}	16.12^{abc}	4.76^{abcdef}	2.48^{abcd}	11.89^{abcde}	6.27^{a}	1.31^{d}	5.65^{abcd}
	L2	2.85^{a}	4.50^{abcde}	11.36^{abcde}	7.82^{abcd}	2.01^{abcde}	8.97^{cdef}	5.60^{ab}	2.90^{bcd}	5.21^{abcd}
	L3	2.50^{a}	1.49^{de}	9.44^{bcde}	4.93^{abcdef}	1.14^{de}	8.97^{cdef}	4.95^{abcd}	3.00^{abcd}	2.29^{d}
	L4	2.80^{a}	0.85^{e}	6.93^{cde}	4.27^{abcdef}	0.86^{de}	7.70^{def}	6.34^{a}	3.79^{abc}	2.27^{d}
	L5	2.85^{a}	0.54^{e}	4.03^{de}	4.24^{abcdef}	0.50^{e}	6.03^{ef}	2.07^{ef}	5.16^{a}	2.17^{d}
	L6	2.87^{a}	4.42^{abcde}	11.32^{abcde}	7.49^{abcde}	1.93^{abcde}	9.14^{cdef}	5.26^{abc}	2.79^{bcd}	5.44^{abcd}
	L7	2.62^{a}	5.35^{abcde}	13.31^{abcde}	6.47^{abcdef}	2.18^{abcde}	10.15^{abcdef}	5.18^{abc}	1.33^{d}	6.25^{abcd}
	L8	2.92^{a}	5.65^{abcde}	13.57^{abcde}	8.27^{abc}	2.22^{abcd}	10.30^{abcde}	5.05^{abc}	1.62^{d}	6.40^{abcd}
	L9	2.26^{a}	6.25^{abcde}	16.49^{abc}	8.92^{a}	2.97^{ab}	10.99^{abcde}	3.77^{bcde}	1.94^{cd}	6.94^{abcd}

Species	Strain	Non-essential amino acid									
		Ala	Arg	Asn	Asp	Glu	Gln	Gly	Pro	Ser	Tyr
–	Control	3.12^{abcde}	31.07^{a}	4.93^{a}	1.29^{abc}	6.83^{cdefg}	0.58^{bc}	1.71^{a}	3.14^{bc}	2.85^{ab}	2.86^{a}
B. subtilis	S1	1.63^{de}	3.98^{cd}	0.26^{b}	0.32^{efg}	1.93^{fg}	1.04^{bc}	0.32^{ef}	6.06^{abc}	0.67^{efgh}	8.00^{ab}
	S2	2.63^{bcde}	12.23^{abcd}	0.13^{b}	0.24^{fg}	2.16^{fg}	0.32^{c}	0.22^{f}	2.11^{c}	0.22^{h}	5.15^{abcde}
	S3	3.25^{abcde}	20.16^{abcd}	0.26^{b}	0.28^{efg}	2.03^{fg}	0.86^{bc}	0.20^{f}	3.64^{bc}	0.25^{h}	5.03^{bcde}
	S4	2.20^{cde}	13.12^{abcd}	0.23^{b}	0.34^{efg}	2.64^{fg}	0.28^{c}	0.20^{f}	7.52^{abc}	0.30^{h}	6.74^{abcd}
	S5	1.97^{de}	2.86^{cd}	0.38^{b}	0.42^{defg}	2.30^{fg}	0.18^{c}	0.77^{def}	12.96^{a}	0.87^{defgh}	8.01^{ab}
	S6	2.81^{bcde}	25.54^{ab}	0.25^{b}	0.31^{efg}	4.40^{defg}	0.16^{c}	0.22^{f}	5.69^{abc}	0.20^{h}	4.65^{cde}
	S7	1.20^{e}	4.24^{cd}	0.34^{b}	0.40^{defg}	2.22^{fg}	0.18^{c}	0.29^{ef}	10.79^{abc}	0.63^{efgh}	8.13^{a}
	S8	1.10^{e}	9.99^{bcd}	0.15^{b}	0.26^{efg}	0.86^{g}	3.24^{a}	0.38^{def}	11.24^{ab}	0.37^{fgh}	7.81^{ab}
	S9	4.44^{abc}	19.20^{abcd}	0.38^{b}	0.30^{efg}	3.10^{efg}	1.76^{b}	0.23^{f}	10.47^{abc}	0.30^{h}	5.29^{abcde}
B. velezensis	V1	3.57^{abcd}	21.06^{abc}	0.51^{b}	0.33^{efg}	5.32^{cdefg}	0.32^{c}	0.21^{f}	4.84^{abc}	0.35^{gh}	6.92^{abcd}
	V2	3.01^{abcde}	11.24^{abcd}	0.69^{b}	0.28^{efg}	3.96^{efg}	0.07^{c}	0.49^{def}	9.27^{abc}	0.79^{defgh}	7.31^{abcd}
	V3	3.85^{abcd}	18.41^{abcd}	0.52^{b}	0.22^{fg}	3.87^{efg}	0.20^{c}	0.17^{f}	5.02^{abc}	0.18^{h}	6.55^{abcd}
	V4	2.33^{cde}	12.96^{abcd}	0.37^{b}	0.37^{efg}	3.54^{efg}	0.29^{c}	0.14^{f}	10.82^{abc}	0.27^{h}	7.29^{abcd}
	V5	2.17^{cde}	3.85^{cd}	0.27^{b}	0.17^{g}	0.67^{g}	0.17^{c}	0.44^{def}	7.46^{abc}	0.53^{efgh}	8.20^{a}
	V6	2.48^{cde}	15.35^{abcd}	0.50^{b}	0.25^{fg}	3.58^{efg}	0.18^{c}	0.22^{f}	5.06^{abc}	0.58^{efgh}	7.25^{abcd}
	V7	2.32^{cde}	9.84^{bcd}	0.72^{b}	0.47^{cdefg}	3.43^{efg}	0.12^{c}	0.28^{ef}	5.53^{abc}	1.45^{cdefg}	7.37^{abcd}
	V8	2.66^{bcde}	16.84^{abcd}	0.76^{b}	0.42^{defg}	4.69^{defg}	0.33^{c}	0.39^{def}	7.64^{abc}	1.48^{cdef}	6.44^{abcd}
	V9	2.49^{cde}	22.31^{abc}	0.47^{b}	0.36^{efg}	4.47^{defg}	0.15^{c}	0.16^{f}	4.43^{abc}	0.43^{fgh}	6.49^{abcd}
B. licheniformis	L1	4.85^{ab}	0.40^{d}	0.32^{b}	1.64^{ab}	16.46^{a}	0.11^{c}	0.95^{bcd}	2.85^{bc}	1.62^{cde}	7.44^{abcd}
	L2	2.67^{bcde}	0.09^{d}	0.51^{b}	0.90^{bcdefg}	10.24^{abcde}	0.21^{c}	0.55^{def}	4.87^{abc}	1.29^{cdefgh}	6.39^{abcd}
	L3	2.75^{bcde}	0.00^{d}	0.72^{b}	1.31^{abc}	17.28^{a}	0.33^{c}	0.52^{def}	2.43^{bc}	0.83^{defgh}	6.08^{abcd}
	L4	3.89^{abcd}	0.00^{d}	0.72^{b}	1.26^{abcd}	12.48^{abc}	0.59^{bc}	0.33^{ef}	2.80^{bc}	1.01^{defgh}	4.67^{cde}
	L5	2.53^{cde}	0.00^{d}	0.62^{b}	0.65^{cdefg}	11.61^{abcd}	0.37^{c}	0.24^{f}	4.72^{abc}	0.51^{efgh}	4.45^{de}
	L6	3.12^{abcde}	0.10^{d}	0.41^{b}	0.75^{cdefg}	8.39^{cdef}	0.31^{c}	0.90^{cde}	5.35^{abc}	1.17^{cdefgh}	6.04^{abcd}
	L7	5.14^{a}	0.40^{d}	0.50^{b}	1.76^{a}	15.87^{ab}	0.42^{c}	1.39^{abc}	4.01^{abc}	2.10^{bc}	6.33^{abcd}
	L8	3.09^{abcde}	0.49^{d}	0.65^{b}	1.14^{abcde}	11.60^{abcd}	0.52^{bc}	0.87^{cde}	8.55^{abc}	1.83^{cd}	6.70^{abcd}
	L9	2.73^{bcde}	0.34^{d}	0.27^{b}	1.10^{abcdef}	9.07^{bcdef}	0.14^{c}	1.48^{ab}	4.41^{abc}	3.12^{a}	7.70^{abc}

Species	Strain	Non-proteinogenic amino acid		
		Cit	GABA	Orn
–	Control	0.10^{a}	15.76^{ab}	0.00^{d}
B. subtilis	S1	0.19^{a}	2.45^{def}	0.17^{d}
	S2	0.23^{a}	9.41^{abcdef}	1.48^{d}
	S3	0.29^{a}	11.51^{abcdef}	0.00^{d}
	S4	1.41^{a}	6.80^{bcdef}	0.00^{d}
	S5	2.47^{a}	0.60^{ef}	0.39^{d}
	S6	1.55^{a}	15.08^{abc}	0.00^{d}
	S7	1.68^{a}	2.49^{def}	0.12^{d}
	S8	0.27^{a}	2.31^{def}	0.70^{d}
	S9	0.60^{a}	12.67^{abcd}	0.78^{d}
B. velezensis	V1	2.09^{a}	11.68^{abcde}	0.00^{d}
	V2	2.93^{a}	5.89^{bcdef}	0.00^{d}
	V3	2.01^{a}	9.47^{abcdef}	0.00^{d}
	V4	1.25^{a}	11.79^{abcde}	2.01^{d}
	V5	1.67^{a}	4.07^{cdef}	0.00^{d}
	V6	3.51^{a}	9.63^{abcdef}	0.00^{d}
	V7	0.80^{a}	4.59^{bcdef}	0.00^{d}
	V8	0.71^{a}	8.03^{abcdef}	0.00^{d}
	V9	1.62^{a}	11.54^{abcdef}	0.00^{d}
B. licheniformis	L1	1.80^{a}	0.91^{ef}	4.83^{cd}
	L2	4.53^{a}	4.87^{bcdef}	11.68^{bc}
	L3	2.60^{a}	9.63^{abcdef}	16.79^{b}
	L4	6.34^{a}	11.32^{abcdef}	18.77^{b}
	L5	2.51^{a}	18.10^{a}	26.10^{a}
	L6	4.54^{a}	6.31^{bcdef}	11.95^{bc}
	L7	2.98^{a}	0.96^{ef}	5.29^{cd}
	L8	3.51^{a}	0.59^{ef}	4.47^{cd}
	L9	1.41^{a}	0.18^{f}	7.53^{cd}

Different superscripts within a column denote a significant difference between mean values ($p<0.05$) according to Duncan's multiple range test.

Fig. S1-1. Scatter plots of Pearson correlation coefficients (PCCs) between the quantities of essential amino acids produced by *B. subtilis* and *B. velezensis* strains in soybean cultures and their proteolytic index (PI) values.

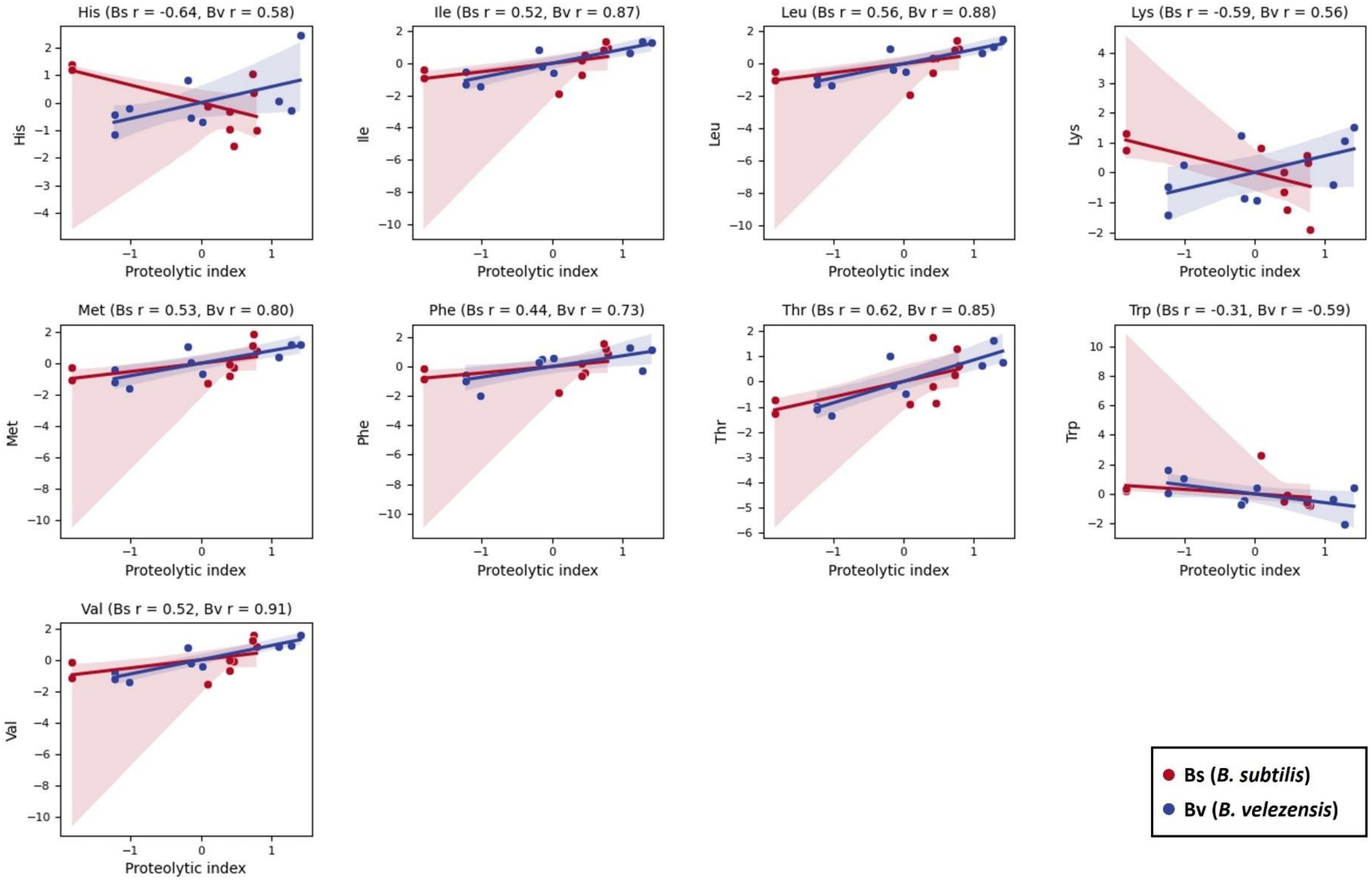

Fig.S1-2. Scatter plots of PCCs between the quantities of non-essential amino acids produced by *B. subtilis* and *B. velezensis* strains in soybean cultures and their PI values.

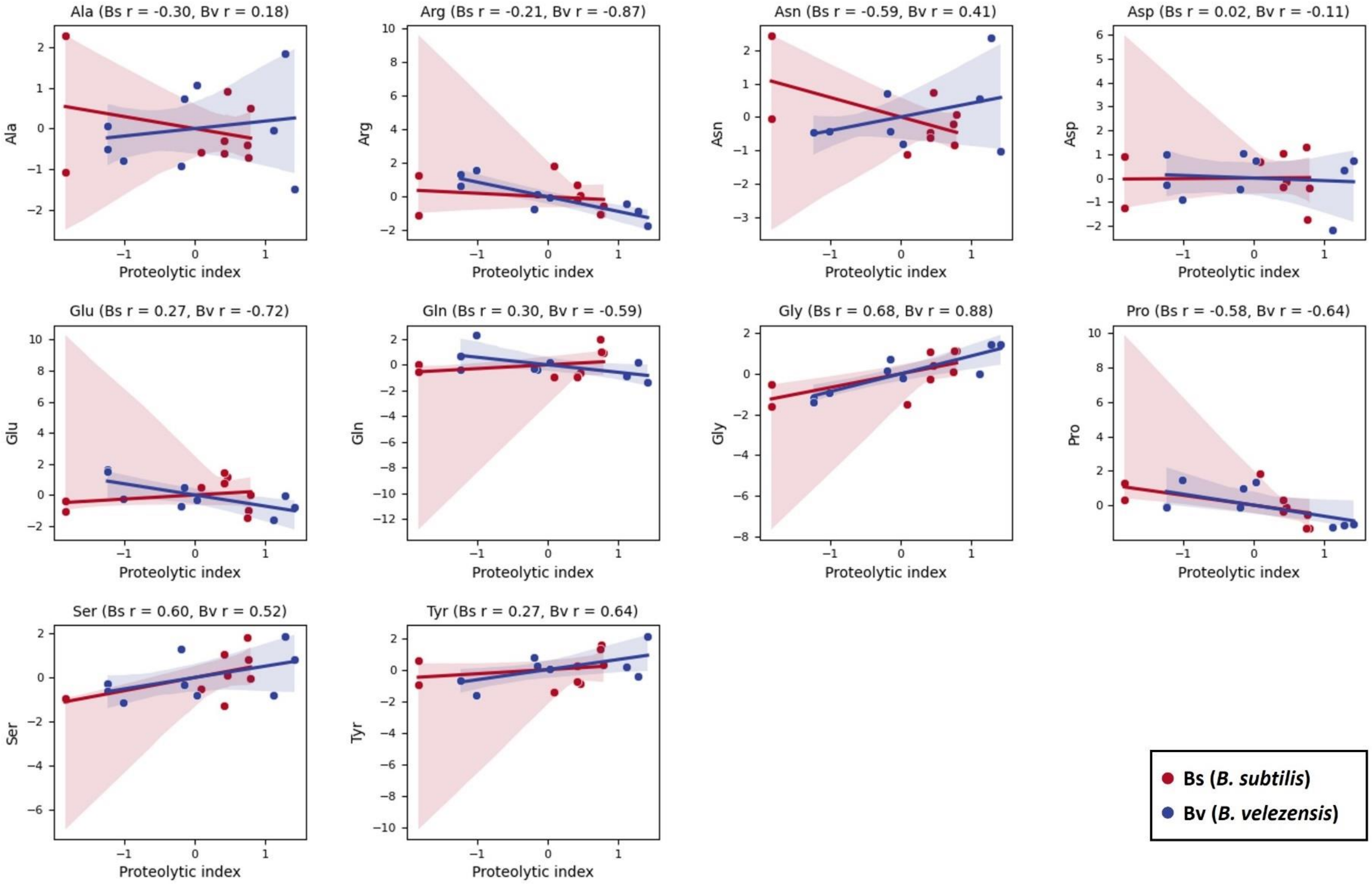

Fig. S1-3. Scatter plots of PCCs between the quantities of non-proteogenic amino acids produced by *B. subtilis* and *B. velezensis* strains in soybean cultures and their PI values.

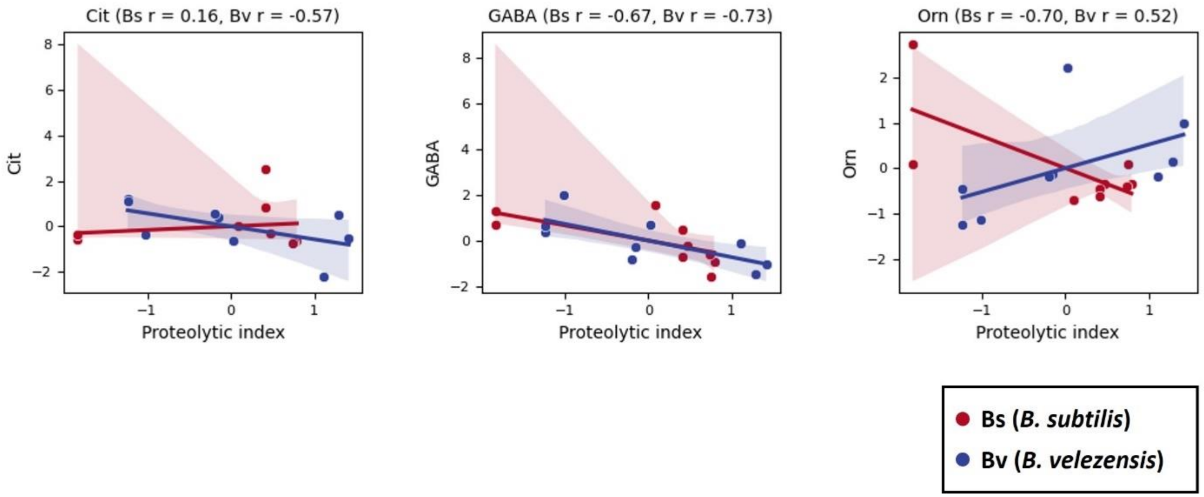

Fig. S2. Average nucleotide identity (ANI) analysis of genomes of *B. subtilis*, *B. velezensis*, and *B. licheniformis*. The published complete genome sequences of 13 *B. subtilis* strains, 15 *B. velezensis* strains, and 8 *B. licheniformis* strains, isolated from fermented soybean foods, were retrieved from NCBI for ANI analysis.

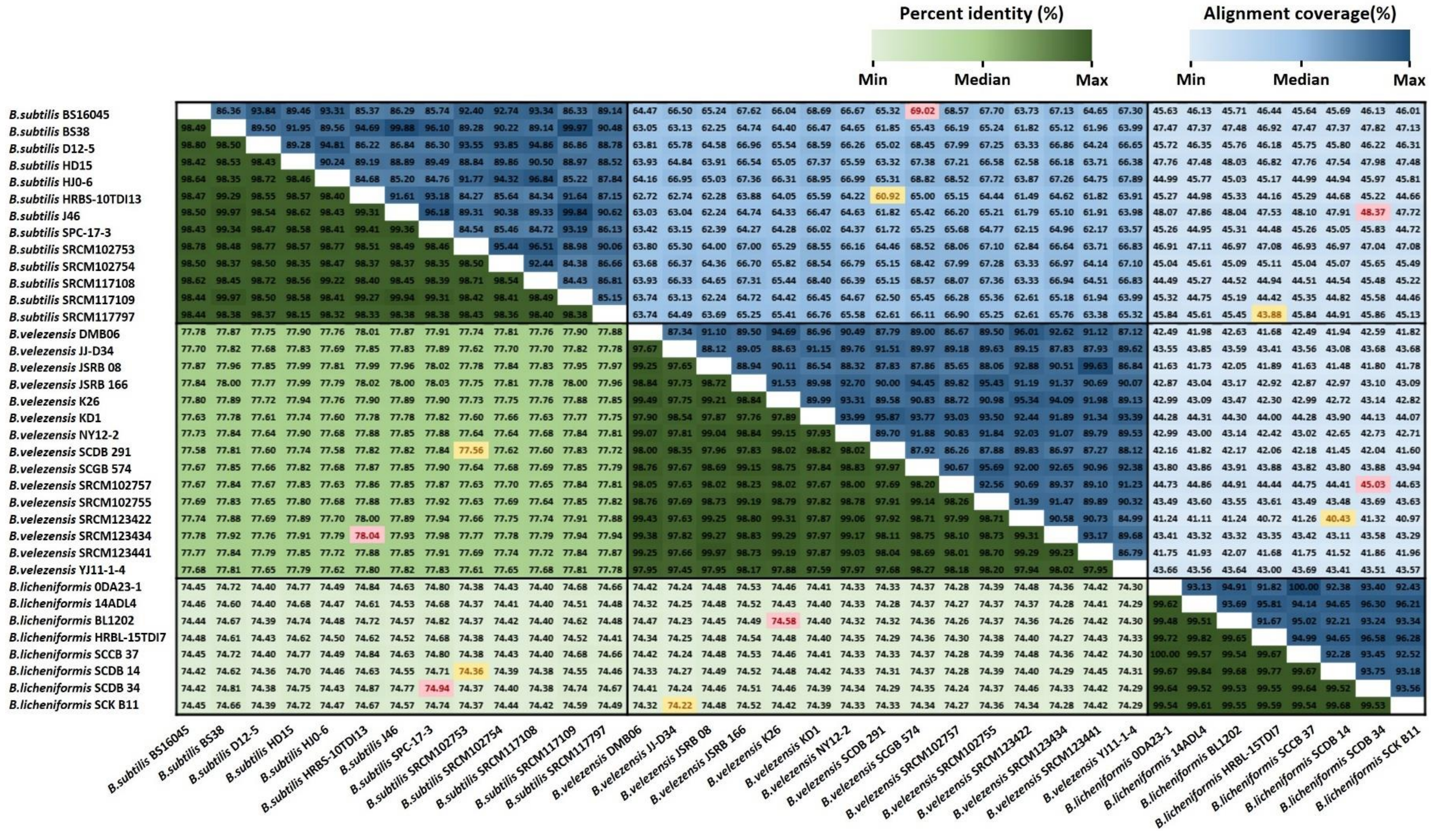

Supplement: Supplementary file 1 [file jmb-35-e2411038-supple.pdf]
